# Major Crop Species Show Differential Balance between Root Morphological and Physiological Responses to Variable Phosphorus Supply

**DOI:** 10.3389/fpls.2016.01939

**Published:** 2016-12-21

**Authors:** Yang Lyu, Hongliang Tang, Haigang Li, Fusuo Zhang, Zed Rengel, William R. Whalley, Jianbo Shen

**Affiliations:** ^1^Centre for Resources, Environment and Food Security, Department of Plant Nutrition, Key Laboratory of Plant-Soil Interactions, Ministry of Education, China Agricultural UniversityBeijing, China; ^2^College of Life Science, Hebei UniversityBaoding, China; ^3^Soil Science and Plant Nutrition, School of Earth and Environment, The UWA Institute of Agriculture, The University of Western Australia, CrawleyWA, Australia; ^4^Rothamsted ResearchHarpenden, UK

**Keywords:** phosphorus uptake, fibrous root species, legume species, root morphological traits, root exudation, phosphorus supply

## Abstract

The relationship between root morphological and physiological responses to variable P supply in different plant species is poorly understood. We compared root morphological and physiological responses to P supply in seven crop species (*Zea mays*, *Triticum aestivum*, *Brassica napus*, *Lupinus albus, Glycine max, Vicia faba*, *Cicer arietinum*) treated with or without 100 mg P kg^-1^ in two soils (acidic and calcareous). Phosphorus deficiency decreased root length more in fibrous root species (*Zea mays, Triticum aestivum, Brassica napus*) than legumes. *Zea mays* and *Triticum aestivum* had higher root/shoot biomass ratio and *Brassica napus* had higher specific root length compared to legumes, whereas legumes (except soybean) had higher carboxylate exudation than fibrous root species. *Lupinus albus* exhibited the highest P-acquisition efficiency due to high exudation of carboxylates and acid phosphatases. *Lupinus albus* and *Cicer arietinum* depended mostly on root exudation (i.e., physiological response) to enhance P acquisition, whereas *Zea mays, Triticum aestivum and Brassica napus* had higher root morphology dependence, with *Glycine max* and *Vicia faba* in between. Principal component analysis using six morphological and six physiological responses identified root size and diameter as the most important morphological traits, whereas important physiological responses included carboxylate exudation, and P-acquisition and P-utilization efficiency followed by rhizosphere soil pH and acid phosphatase activity. In conclusion, plant species can be grouped on the basis of their response to soil P being primarily via root architectural or exudation plasticity, suggesting a potential benefit of crop-specific root-trait-based management to cope with variable soil P supply in sustainable grain production.

## Introduction

Phosphorus (P) is an essential macronutrient for plant growth and metabolism. It is a structural element in nucleic acids and membrane phospholipids. However, P nutrition is a major limiting factor for crop production in many soils due to relatively low P availability because P can be readily adsorbed or fixed by free lime present in some calcareous soils (CS) and by aluminium (Al) and iron (Fe) in acid soils (AS) (Hinsinger, 2001; Shen et al., 2011). Around 70–90% of P applied as fertilizer may become unavailable to plants (Holford, 1997), exacerbating economic losses from fertilizer-P overuse in intensive agriculture (Shen et al., 2011). On the other hand, P is a limited and non-renewable resource; current estimates suggest that economic P supply may be severely depleted over the next 300 years, although some research indicates that estimates of P reserves have increased by more than threefold recently (Oelkers and Valsami-Jones, 2008; Cordell et al., 2011; Elser and Bennett, 2011; Cordell and White, 2013). Improvement of P-acquisition efficiency through mobilizing the residual P accumulated in soil, as well as enhancing root absorbing surface and acquisition capacity for P applied to soil, is critical for sustainable P management and food production (Shen et al., 2011, 2013; Li et al., 2014).

In response to low concentration of available P in the rhizosphere, plants have developed highly specialized root morphological (e.g., increases in root growth rate, specific root length (SRL), lateral density and elongation, and density and length of root hairs) and physiological (e.g., carboxylate exudation, proton release, and phosphatase secretion) adaptations to increase P acquisition from soil (Neumann et al., 1999; Hinsinger, 2001; Lambers et al., 2006; Shen et al., 2011). The ultimate consequence of these modifications is increased P availability and acquisition via (1) increasing a rhizosphere soil volume exploited by an enlarged root length/surface area to improve soil P spatial availability, and (2) enhancing P availability by mobilizing P in the rhizosphere via root exudation (Raghothama, 1999; Rengel and Marschner, 2005; Richardson et al., 2009; Shen et al., 2011).

Many studies showed a range of adaptive strategies (especially in terms of root growth and rhizosphere processes) evolved to cope with limited P availability and allow efficient P acquisition by different plant species (Lambers et al., 2006; Zhang et al., 2010; Shen et al., 2011, 2013). For example, P-deficient *Brassica napus* had high P influx rates, whereas P-deficient *Triticum aestivum* had high root/shoot ratios to enhance P-acquisition efficiency (Föhse et al., 1988). Under low P supply, the root/shoot ratio increased in *Zea mays* and *Triticum aestivum* significantly, and *Triticum aestivum* had increased SRL (Nuruzzaman et al., 2005; Pearse et al., 2006a, 2007; Calderón-Vázquez et al., 2009). In contrast, there was no difference in root carboxylate exudation by *Zea mays* or *Triticum aestivum* between P-sufficient and P-deficient plants (Pearse et al., 2006b, 2007; Carvalhais et al., 2011). However, for *Brassica napus*, P deficiency increased the root hair density and length and enhanced exudation of protons and carboxylates (Foehse and Jungk, 1983; Moorby et al., 1988; Hoffland et al., 1989a,b). The exudation of acid phosphatase by *Brassica napus* increased with increasing P supply (Marschner et al., 2006, 2007; Solaiman et al., 2007; Zhang et al., 2009).

Legume plants enhance rhizosphere chemical processes more than cereal crops to mobilize sparingly soluble soil P by rhizosphere acidification and enhanced exudation of carboxylates and phosphatases (Houlton et al., 2008; Shen et al., 2013; Li et al., 2014). For example, *Lupinus albus* could respond to P deficiency stress by forming cluster roots (Gardner et al., 1982) accompanied by high exudation of carboxylates, protons and acid phosphatase from such roots, which greatly enhanced P acquisition from soil (Tadano et al., 1993; Neumann et al., 1999; Yan et al., 2002; Shen et al., 2003; Vance et al., 2003; Lambers et al., 2006; Wang et al., 2007; Cheng et al., 2014). In addition, P uptake by *Cicer arietinum* exhibited a positive correlation with rate of carboxylate exudation into the rhizosphere (Veneklaas et al., 2003; Wouterlood et al., 2004; Rose et al., 2010) as well as with the activity of acid phosphatase (APase) extruded by roots (Li et al., 2004; Pearse et al., 2006b, 2007). Phosphorus deficiency strongly increased proton release from roots of tomato, chickpea, and white lupin, but only small effects were observed in wheat (Neumann and Römheld, 1999). Compared with white lupin, root exudation of carboxylates under P deficiency was lower in tomato, wheat and chickpea (Neumann and Römheld, 1999).

Soil acid phosphatase activity was higher in the rhizosphere of *Cicer arietinum* than *Zea mays* regardless of P sources (Li et al., 2004). While root morphological traits in *Cicer arietinum* had a minor contribution to potentially enhancing P uptake in the low-P environments, the concentration of carboxylates in the rhizosphere increased 10-fold (Veneklaas et al., 2003). Under P-deficient conditions, roots of *Glycine max* exuded more carboxylates than *Zea mays*, but much less than *Cicer arietinum, Vicia faba*, and *Lupinus albus* (Ohwaki and Hirata, 1992; Watt and Evans, 2003; Li et al., 2007). *Vicia faba* released smaller amounts of protons and carboxylates into rhizosphere than *Cicer arietinum*, but much greater than *Triticum aestivum* and *Zea mays* (Zhou et al., 2009; Li et al., 2010; Rose et al., 2010). In Maltais-Landry’s (2015) study, legumes (*Vicia faba* and *Pisum sativum*) had higher organic acid concentration and phosphatase activity in the rhizosphere compared with cereals crops (*Secale cereale*, *Avena sativa*, and *Triticum aestivum*).

To reiterate, plants can enhance phosphorus (P) acquisition from soil via modifying root morphological and physiological traits. We assumed that some plant species were mainly dependent on the enhanced root growth and spatial distribution of roots, whereas others could dominantly rely on increased root exudation to mobilize soil P; a strategy combining both types of responses is also possible. Understanding the complexity of the relationships between root morphological and physiological responses across different plant species is critical for improved manipulation of the root and rhizosphere processes to increase P-acquisition efficiency for a given plant species.

In this study, we hypothesized an existence of different combinations of strategies related to root morphological and physiological adaptations to cope with variable P supply in seven plant species with contrasting root systems. We intended to determine if plant species could be grouped based on their response to soil P being predominantly via root architectural plasticity (morphological response) or exudation (physiological response). We chose seven plant species widely grown in agriculture, having either fibrous (*Zea mays*, *Triticum aestivum*, and *Brassica napus*) or tap-rooted root systems (*Lupinus albus, Glycine max, Vicia faba*, and *Cicer arietinum*) to test the above hypothesis. Our objective was to characterize the relationship between root morphological and physiological responses to P supply in different plant species with contrasting root systems in AS or CS.

## Materials and Methods

### Plant and Soil Materials

Seven plant species were tested in the study, including those with fibrous roots (*Zea mays* L. cv. NE15 (Zm), *Triticum aestivum* L. cv. KN9204 (Ta), *Brassica napus* L. cv. LY5 (Bn)) and tap-rooted legumes (*Lupinus albus* L. Kiev mutant (La), *Glycine max* (L.) Merr. cv. HX1 (Gm), *Vicia faba* L. cv. LC5 (Vf) and *Cicer arietinum* L. cv. LY1 (Ca)). In this study, *Brassica napus* was factitiously regarded as a fibrous root species due to its fine root system based on our previous study (Solaiman et al., 2007).

Acid soil and CS with low P availability were collected from the top 20 cm of unfertilized native vegetation sites: AS in Guangdong, South China (23° 27′ 21″ N, 114° 31′ 39″ E), and CS in Beijing area, North China (40° 8’ 5? N, 116° 11’ 3? E). AS had the following properties (in parentheses: CS): pH 5.5 (8.2) in 1:5 soil:CaCl_2_, organic carbon 14.6 (11.5) g kg^-1^, total N 0.37 (0.72) g kg^-1^, Olsen-P 7.1 (2.6) mg kg^-1^, and NH_4_Ac-extractable K 44 (32) mg kg^-1^. The soils were air-dried and sieved to 2 mm prior to potting. Field capacity moisture contents were determined to be 35% w/w for AS and 32% w/w for CS according to Rose et al. (2010).

### Plant Growth

The experiment was conducted in a glasshouse at China Agricultural University, Beijing (40° 1′ 46″ N, 116° 17′ 11″ E). Plants were grown in 240-mm-wide and 190-mm-deep plastic pots containing 3 kg soil each. Phosphorus was added to soils as KH_2_PO_4_ at a rate of 100 (P-sufficient) or 0 (P-deficient) mg P kg^-1^ soil. All other basal nutrients were provided as follows (mg per pot): Ca(NO3)2⋅4H2O 5060; K_2_SO_4_ 400; CaCl_2_ 377; MgSO_4_⋅7H_2_O 130; EDTA-Fe 16.5; MnSO_4_⋅H_2_O 20; ZnSO_4_⋅7H_2_O 30; CuSO_4_⋅5H_2_O 6; H_3_BO_3_ 2; and (NH_4_)_6_Mo_7_O_24_⋅4H_2_O 0.365. There were four replicates in each treatment. Soils were irrigated to approximately 80% field capacity with deionized water prior to sowing.

Seeds of all species were surface-sterilized by 10% v/v H_2_O_2_ for 30 min, rinsed with water and placed in a dish containing aerated saturated solution of CaSO_4_ at 26°C in the dark until a radicle emerged. Six germinated seeds of uniform size were sown in each pot. Seedlings were thinned to four plants per pot 5 days after germination. Each pot was watered daily to 80% field capacity as measured by weight. Temperature ranged from a minimum of 22°C at night to a maximum of 30°C during the day.

### Plant Harvest and Root Sampling

**FIGURE 1:**
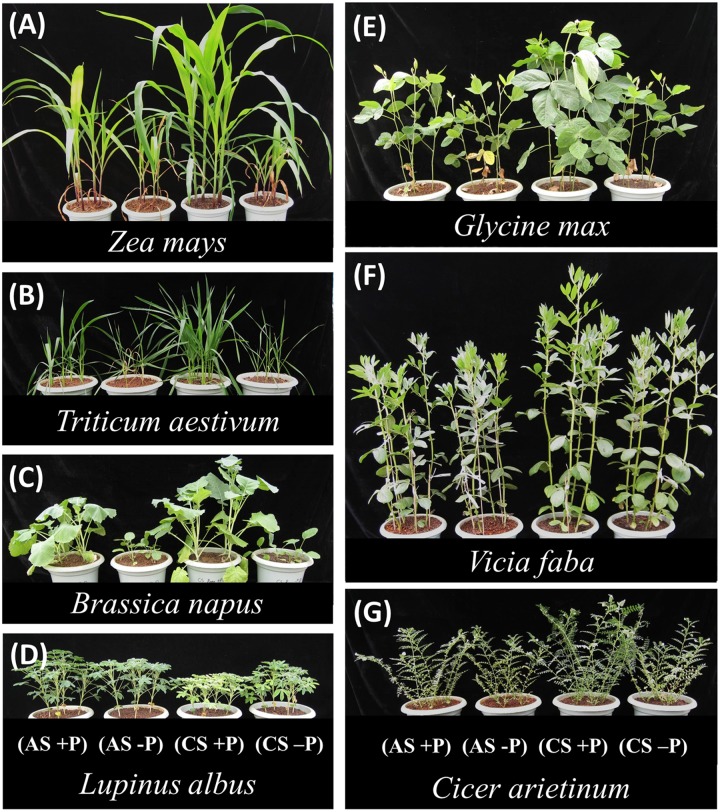
**Shoot growth of seven species after 40 days (just before harvest).** For each species, the treatments are (from left to right): acid soil (AS) with P addition (100 mg P kg^-1^ soil) (AS+P), AS without P addition (AS-P), calcareous soil (CS) with P addition (100 mg P kg^-1^ soil) (CS+P) and CS without P addition (CS-P).

Plants were harvested 40 days after germination; at this time visual differences in growth between P treatments or soil types could be observed (**Figure 1**). The method for rhizosphere exudate collection was modified from Pearse et al. (2007). The pots were squeezed gently to allow dislodgement of the soil column and loosening of soil around roots. Plants were then gently lifted from soil and shaken lightly to remove bulk soil from the root systems. The root system was then transferred into a 200-mL vial containing 50 mL of 0.2 mmol L^-1^ CaCl_2_. Roots were gently dunked for 60 s to remove as much rhizosphere soil as possible; care was taken to minimize root damage and thus leakage of solutes from damaged cells. After removing roots, the containers were shaken by hand, and 0.5 mL of soil suspension was transferred into a 2-mL centrifuge tube for measurement of acid phosphatase (APase) activity (Alvey et al., 2001), representing secretory acid phosphatase (Neumann et al., 1999; Shen et al., 2005). A sub-sample of 10 mL supernatant from soil suspension was kept in a vial [with addition of microbial inhibitor Micropur (Sicheres Trinkwasser, Germany) at 0.01 g L^-1^ and also three drops of concentrated phosphoric acid] at –20°C until analysis of carboxylates by HPLC that was done after passing the supernatant through a 0.22-μm filter according to the method developed in our lab (Shen et al., 2003; Wang et al., 2007).

### Root Parameter Measurement

The roots from each pot were washed out of soil with water and evenly spread apart on a transparent tray (25 cm × 19 cm) to get images at a resolution of 600 dpi (dots per inch) with an Epson Perfection V700 dual lens scanning system. Root images were analyzed for total root length and total root surface area using WinRHIZO software (Pro 2009b, Regent Instruments Inc., Quebec, QC, Canada).

### Plant Biomass and Phosphorus Uptake

Shoots and roots were oven-dried at 70°C for 3 days until constant weight to measure biomass. Shoots and roots were ground into powder, and then weighed and digested with a mixture of concentrated H_2_SO_4_ and H_2_O_2_ (modified from Thomas et al., 1967). Phosphorus content was determined using the vanado-molybdate method (Westerman, 1990).

Phosphorus-acquisition efficiency refers to the ability of plants to acquire P from soils, and P-utilization efficiency is the capacity to produce biomass or yield using the P taken up (Hammond et al., 2009; Wang X.R. et al., 2010). In this study, P-acquisition efficiency was calculated by dividing the P content of whole plant (shoots + roots) by total root length; P-utilization efficiency was calculated by dividing the total plant biomass by whole-plant P content.

### Acid Phosphatase Activity and Carboxylate Analysis

Acid phosphatase activity in the rhizosphere was measured using a spectrophotometric method based on the measurement of *p*-nitrophenol (PNP) absorbance at 405 nm (Alvey et al., 2001). Carboxylates in the rhizosphere soil were analyzed using a reversed phase high-performance liquid chromatography (HPLC) system according to a previous report (modified from Shen et al., 2003; Wang et al., 2007; Wang B.L. et al., 2010). The chromatographic separation was conducted on a 250 mm × 4.6 mm reversed-phase column (Alltima C18, 5 μm; Alltech Associates, Inc., Deerfield, IL, USA). The mobile phase was 25 mmol L^-1^ KH_2_PO_4_ (pH 2.25) with a flow rate of 1 mL min^-1^ at 31°C, and detection of carboxylates was carried out at 214 nm.

### Statistical Analyses

Analysis of variance was conducted using the SAS statistical software (SAS 2001, Version 6.1, SAS Institute Inc., USA). The LSD multiple range comparisons were performed at the 5, 1, and 0.1% probability level (0.01 < *P* ≤ 0.05, 0.001 < *P* ≤ 0.01, and *P* ≤ 0.001).

Principal component analysis (PCA) was used to evaluate the relative responses of root morphological and physiological traits to P deficiency in two soil types based on our previous method (Tang et al., 2013b). Six root morphological variables (total root surface area, root biomass, total root length, root/shoot ratio, SRL, and specific root surface area) and six root physiological variables (malate exudation, citrate exudation, P-acquisition efficiency, acid phosphatase activity, rhizosphere pH, P-utilization efficiency) were analyzed. We did not use the root clusters as a morphological parameter in this PCA analysis because only one (*Lupinus albus*) out of seven species tested had these special root structures (**Supplementary Figure S1**). The first three principal components were used to describe the relative responses of seven plant species to P deficiency in two soil types and to calculate the total scores for root morphological or physiological response variables.

## Results

### Biomass Accumulation and Distribution

**FIGURE 2:**
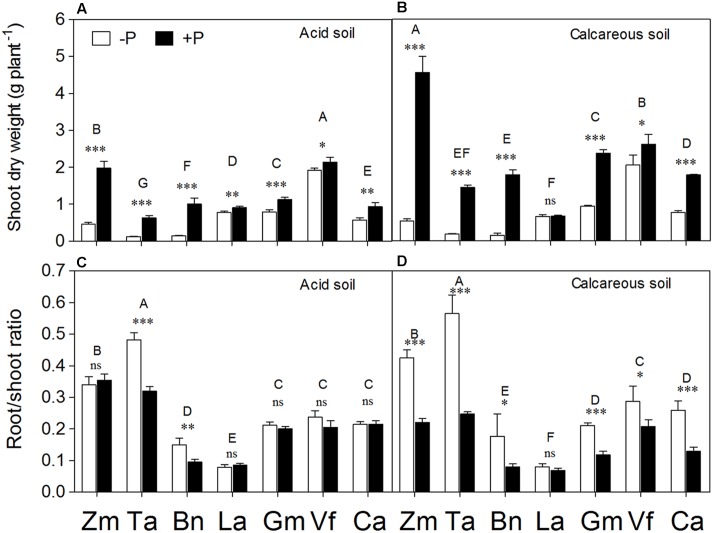
**Shoot dry weight** **(A,B)** and root/shoot ratio **(C,D)** of *Zea mays* (Zm), *Triticum aestivum* (Ta), *Brassica napus* (Bn), *Lupinus albus* (La), *Glycine max* (Gm), *Vicia faba* (Vf), and *Cicer arietinum* (Ca) supplied with 0 (open bars) or 100 mg P kg^-1^ soil (closed bars) in AS **(A,C)** or CS **(B,D)**. Each value is the mean (+SE) of four replicates. Different letters denote significant differences among seven plant species (*P* ≤ 0.05). For a given species, asterisks indicate significant difference between the P treatments: ns (non-significant), ^∗^(0.01 < *P* ≤ 0.05),^∗∗^(0.001 < *P* ≤ 0.01), and ^∗∗∗^(*P* ≤ 0.001).

The ANOVA analysis showed that shoot dry weight varied among species depending on differential P supply and soil types (**Supplementary Table S1**). Phosphorus supply significantly stimulated shoot growth of all species, except *Lupinus albus* in CS (**Figures 2A,B**). The magnitude of shoot dry weight response to P application in AS followed the order *Brassica napus* (+598%) > *Triticum aestivum* (+410%) > *Zea mays* (+325%) > *Cicer arietinum* (+63%) > *Glycine max* (+44%) > *Lupinus albus* (+17%) > *Vicia faba* (+11%), and the order in the CS was *Brassica napus* (+1093%) > *Zea mays* (+749%) > *Triticum aestivum* (+704%) > *Glycine max* (+155%) > *Cicer arietinum* (+131%) > *Vicia faba* (+27%) > *Lupinus albus* (+2%). The fibrous root species *Zea mays*, *Triticum aestivum*, and *Brassica napus* exhibited greater shoot growth responses to P supply than the tap-rooted legumes *Lupinus albus, Glycine max, Vicia faba*, and *Cicer arietinum* regardless of soil type. However, the general response of shoot dry weight to P application was greater in CS than AS (*P* < 0.01), despite a variation among different plant species. In AS, the species accumulated shoot biomass in the order *Vicia faba* > *Zea mays* > *Glycine max* > *Lupinus albus* > *Cicer arietinum* > *Brassica napus* > *Triticum aestivum.* In CS, the order was *Zea mays* > *Vicia faba* > *Glycine max* > *Cicer arietinum* > *Brassica napus* > *Triticum aestivum* > *Lupinus albus*.

*Zea mays* and *Triticum aestivum* had higher root/shoot ratio relative to other species in both soils (**Figures 2C,D**). *Lupinus albus* had the lowest root/shoot ratio among all the species, with a relatively weak response to differential P supply in both soils. In CS, P deficiency significantly enhanced the root/shoot ratio of plant species, except in *Lupinus albus* (**Figures 2C,D**). The fibrous root species *Zea mays*, *Triticum aestivum*, *and Brassica napus* growing in CS exhibited more evident effects of P deficiency on increasing root/shoot ratio compared with the tap-rooted legume species *Vicia faba*, *Glycine max*, *Lupinus albus*, and *Cicer arietinum*. In contrast, in AS, the low-P conditions significantly enhanced root/shoot ratio only in *Triticum aestivum and Brassica napus*. Nevertheless, there were no significant differences in root/shoot ratio due to the soil factor (**Supplementary Table S1**).

### Root Morphology

**FIGURE 3:**
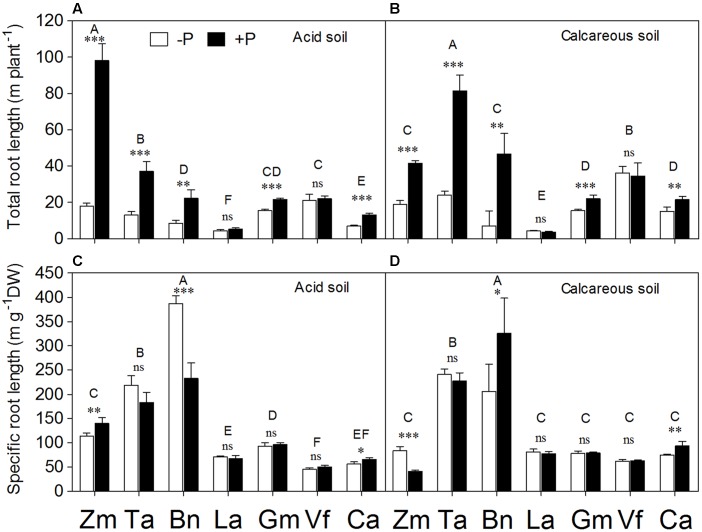
**Total root length** **(A,B)** and specific root length (SRL) **(C,D)** of *Zea mays* (Zm), *Triticum aestivum* (Ta), *Brassica napus* (Bn), *Lupinus albus* (La), *Glycine max* (Gm), *Vicia faba* (Vf), and *Cicer arietinum* (Ca) supplied with 0 (open bars) or 100 mg P kg^-1^ soil (closed bars) in AS **(A,C)** or CS **(B,D)**. Each value is the mean (+SE) of four replicates. Different letters denote significant differences among seven plant species (*P* ≤ 0.05). For a given species, asterisks indicate significant difference between the P treatments: ns (non-significant), ^∗^(0.01 < *P* ≤ 0.05),^∗∗^(0.001 < *P* ≤ 0.01), and ^∗∗∗^(*P* ≤ 0.001).

The fibrous root species *Zea mays*, *Triticum aestivum*, and *Brassica napus* exhibited greater responses of total root length to P supply compared with the tap-rooted legumes *Lupinus albus, Glycine max, Vicia faba*, and *Cicer arietinum* in both soils (**Figures 3A,B**). In AS, total root length per plant was in the order of *Zea mays* > *Triticum aestivum* > *Vicia faba* > *Glycine max* > *Brassica napus* > *Cicer arietinum* > *Lupinus albus*. In CS, the order was *Triticum aestivum* > *Vicia faba* > *Zea mays* = *Brassica napus* > *Glycine max* = *Cicer arietinum* > *Lupinus albus*. Phosphorus application significantly enhanced total root length of *Zea mays*, *Triticum aestivum, Brassica napus, Glycine max* and *Cicer arietinum*, but not of *Lupinus albus* and *Vicia faba* in the two soil types.

Specific root length (ratio of total root length to root biomass) showed significant differences among species, but not between soil types, or P levels (**Supplementary Table S1**). In both soils, *Brassica napus* had the highest SRL, followed by *Triticum aestivum* (**Figures 3C,D**). Phosphorus deficiency significantly increased SRL of *Brassica napus* in AS, but a reverse occurred in CS. In contrast, P deficiency significantly increased SRL of *Zea mays* in CS, but decreased it in AS. *Cicer arietinum* exhibited a higher SRL in the +P than –P treatments in both soils. There was no significant difference in SRL between the P treatments in *Triticum aestivum*, *Lupinus albus*, *Glycine max*, and *Vicia faba* regardless of the soil.

### Acid Phosphatase Activity

**FIGURE 4:**
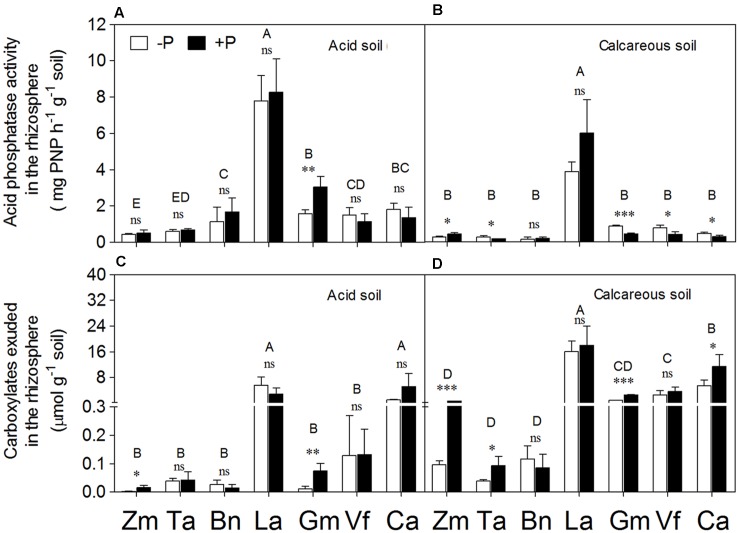
**Acid phosphatase activity** **(A,B)** and total carboxylate concentration **(C,D)** in the rhizosphere of *Zea mays* (Zm), *Triticum aestivum* (Ta), *Brassica napus* (Bn), *Lupinus albus* (La), *Glycine max* (Gm), *Vicia faba* (Vf), and *Cicer arietinum* (Ca) supplied with 0 (open bars) or 100 mg P kg^-1^ soil (closed bars) in AS **(A,C)** or CS **(B,D)**. Each value is the mean (+SE) of four replicates. Different letters denote significant differences among seven plant species (*P* ≤ 0.05). For a given species, asterisks indicate significant difference between the P treatments: ns (non-significant), ^∗^(0.01 < *P* ≤ 0.05),^∗∗^(0.001 < *P* ≤ 0.01), and ^∗∗∗^(*P* ≤ 0.001).

*Lupinus albus* had the highest APase activity among plant species (on average 3.5 and 7.6 times higher in AS and CS, respectively), but there was no evident response of *Lupinus albus* to P application (**Figures 4A,B**). In AS, P deficiency did not change the activity of APase in the rhizosphere of any of the species except *Glycine max* in which an increase occurred with P application. In CS, *Triticum aestivum*, *Glycine max*, *Vicia faba*, and *Cicer arietinum* exhibited significantly higher activity of APase under P deficiency relative to the P-sufficient treatment, but *Zea mays* showed an opposite response.

### Carboxylate Exudation into the Rhizosphere

Significantly higher amounts of carboxylates were measured in the rhizosphere of *Lupinus albus* and *Cicer arietinum* relative to the other five species in AS (**Figures 4C,D**). A similar trend was found in CS, with the tap-rooted legumes *Lupinus albus*, *Vicia faba*, and *Cicer arietinum* releasing a larger amount of carboxylates to the rhizosphere than the fibrous-root species *Zea mays*, *Triticum aestivum*, and *Brassica napus*.

Compared to the P-sufficiency treatment, P-deficiency stress did not increase carboxylate exudation into the rhizosphere in either soil type. On the contrary, P application stimulated carboxylate exudation in *Zea mays* and *Glycine max* in both soils and in *Triticum aestivum* and *Cicer arietinum* in CS.

**FIGURE 5:**
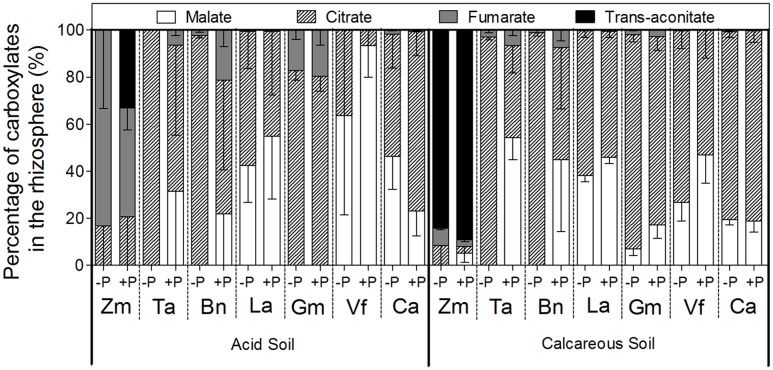
**Proportion of various carboxylates in the rhizosphere soil of *Zea mays* (Zm), *Triticum aestivum* (Ta), *Brassica napus* (Bn), *Lupinus albus* (La), *Glycine max* (Gm), *Vicia faba* (Vf), and *Cicer arietinum* (Ca) supplied with 0 (–P) or 100 mg P kg^-1^ soil (+P) in AS or CS.** Each value is the mean (+SE) of four replicates.

Carboxylate composition varied among species. Malate and citrate were the two major carboxylates in the rhizosphere of all the species except *Zea mays* (**Figure 5**). In CS, *Zea mays* was the only species that not just exuded trans-aconitate, but exuded it as the major carboxylate (on average, 88% of total). When *Zea mays* was grown in AS, *trans*-aconitate was not found in the -P treatment, but represented 33% of all carboxylates in the +P treatment, whereas fumarate was 83% of all carboxylates in the P-deficient treatment and almost 50% in the P-sufficient treatment. *Triticum aestivum*, *Brassica napus*, *Lupinus albus*, *Glycine max*, and *Vicia faba* increased the percentage of citrate in root exudates under P deficiency compared with the +P treatment in both soil types. On the contrary, in AS, *Cicer arietinum* decreased the proportion of citrate exuded under P deficiency, but increased malate exudation compared with CS.

### Shoot P Concentration and Content

**FIGURE 6:**
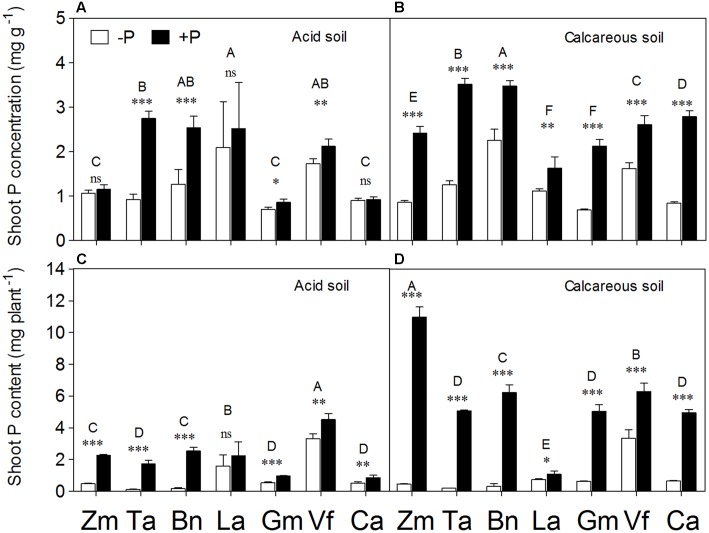
**Phosphorus concentration** **(A,B)** and content **(C,D)** in the shoot tissues of *Zea mays* (Zm), *Triticum aestivum* (Ta), *Brassica napus* (Bn), *Lupinus albus* (La), *Glycine max* (Gm), *Vicia faba* (Vf), and *Cicer arietinum* (Ca) supplied with 0 (open bars) or 100 mg P kg^-1^ soil (closed bars) in AS **(A,C)** or CS **(B,D)**. Each value is the mean (+SE) of four replicates. Different letters denote significant differences among seven plant species (*P* ≤ 0.05). For a given species, asterisks indicate significant difference between the P treatments: ns (non-significant), ^∗^(0.01 < *P* ≤ 0.05),^∗∗^(0.001 < *P* ≤ 0.01), and ^∗∗∗^(*P* ≤ 0.001).

Phosphorus application significantly increased shoot P concentration in all species × soil combinations, except *Zea mays*, *Lupinus albus*, and *Cicer arietinum* in AS (**Figures 6A,B**). The effect of P supply on increased shoot P concentration became more evident in calcareous than AS. Similar to shoot P concentration, the +P treatment significantly increased shoot P content in all species in two soils, except *Lupinus albus* in AS (**Figures 6C,D**). In particular, huge differences in shoot P content were noted in CS, with 23–24 times greater P content in shoots of *Zea mays* and *Triticum aestivum* in the +P than –P treatments.

### P-Acquisition and P-Utilization Efficiency

**FIGURE 7:**
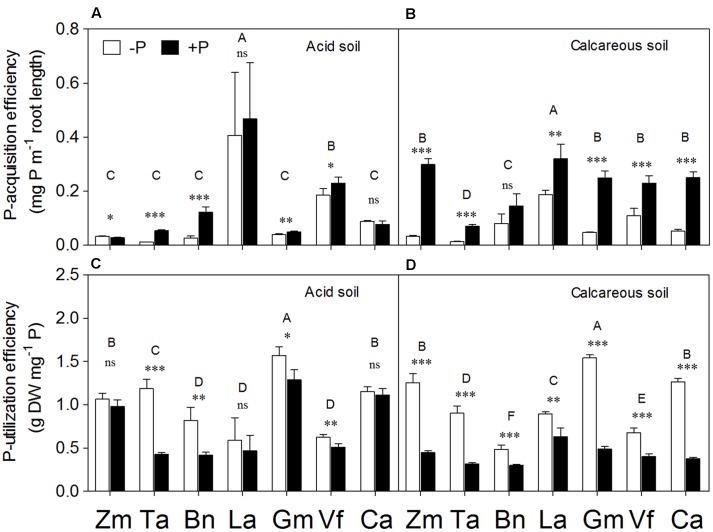
**Phosphorus-acquisition efficiency** **(A,B)** and P-utilization efficiency **(C,D)** of *Zea mays* (Zm), *Triticum aestivum* (Ta), *Brassica napus* (Bn), *Lupinus albus* (La), *Glycine max* (Gm), *Vicia faba* (Vf), and *Cicer arietinum* (Ca) supplied with 0 (open bars) or 100 mg P kg^-1^ soil (closed bars) in AS **(A,C)** or CS **(B,D)**. Each value is the mean (+SE) of four replicates. Different letters denote significant differences among seven plant species(*P* ≤ 0.05). For a given species, asterisks indicate significant difference between the P treatments: ns (non-significant), ^∗^(0.01 < *P* ≤ 0.05),^∗∗^(0.001 < *P* ≤ 0.01), and ^∗∗∗^ (*P* ≤ 0.001).

Plant species and P supply had the significant effects on P-acquisition and P-utilization efficiency (**Supplementary Table S1**). *Lupinus albus* had the highest P-acquisition efficiency among all species in both soil types (**Figures 7A,B**). In general, all species had a significant increase in P-acquisition efficiency in the +P relative to P-deficiency treatment, except *Lupinus albus* and *Cicer arietinum* in AS and *Brassica napus* in CS. In contrast, P deficiency significantly increased P-utilization efficiency in all species compared with the +P treatment, except *Zea mays*, *Lupinus albus*, and *Cicer arietinum* in AS.

### Root Morphological and Physiological Responses

For the root morphological responses, the first principal component (PC1) was related to root size and accounted for 45% of the total variance. Principal component 2 was root diameter (root fineness), explaining 33% of the total variance. Principal component 3 was root/shoot ratio, accounting for 15% of total variance. For the root physiological responses, the PC 1 was related to carboxylate exudation into the rhizosphere, explaining 35% of the total variance. The PC 2 represented P-acquisition and P-utilization efficiency, accounting for 28% of the total variance. The PC 3 comprised rhizosphere soil pH and acid phosphatase activity, accounting for 22% of total variance (**Supplementary Table S2**).

**FIGURE 8:**
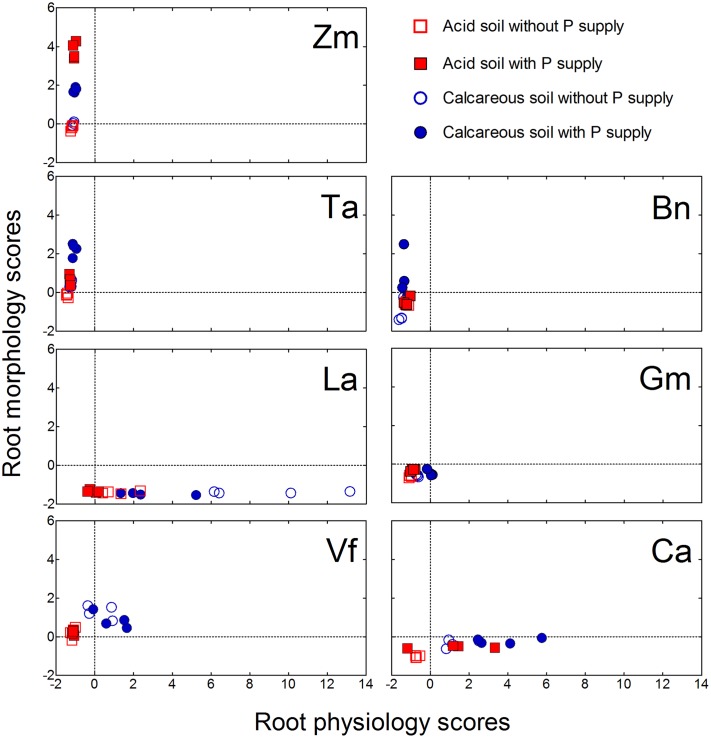
**Principal components analysis of root morphological and physiological responses to variable phosphorus supply and soil types in *Zea mays* (Zm), *Triticum aestivum* (Ta), *Brassica napus* (Bn), *Lupinus albus* (La), *Glycine max* (Gm), *Vicia faba* (Vf), and *Cicer arietinum* (Ca).** Each symbol represents one replicate.

All plant species showed a wide variation in morphological and physiological responses to variable soil P supply (**Figure 8**). *Zea mays* grown in both soils had the root morphology scores significantly increased with P addition, whereas root physiology scores did not show differences. A similar trend was also found in *Triticum aestivum* and *Brassica napus*. In contrast to the fibrous-root species, *Lupinus albus* and *Cicer arietinum* had significant differences in root physiology scores and nearly no differences in root morphology scores with differential P supply. *Glycine max* and *Vicia faba* did not show significant differences in either morphological or physiological scores with differential P supply.

**FIGURE 9:**
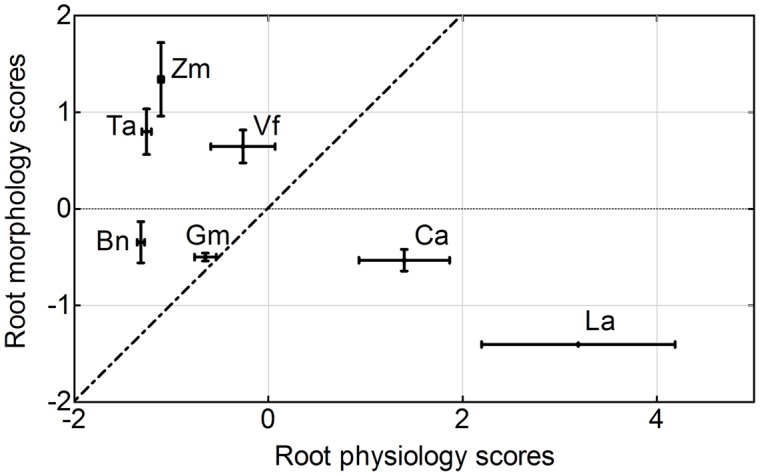
**Principal components analysis of root morphological and physiological responses to variable phosphorus supply by *Zea mays* (Zm), *Triticum aestivum* (Ta), *Brassica napus* (Bn), *Lupinus albus* (La), *Glycine max* (Gm), *Vicia faba* (Vf), and *Cicer arietinum* (Ca) supplied with 0 or 100 mg P kg^-1^ soil in AS or CS.** Each value is the mean (±SE in each direction) of 16 replicates for each plant species. A dashed line represents root morphology scores equal to root physiology scores.

There were large differences in average root morphology and physiology scores among the plant species. *Zea mays* had the highest root morphology scores, followed by *Triticum aestivum* = *Vicia faba* > *Brassica napus* = *Glycine max* = *Cicer arietinum* > *Lupinus albus*. In contrast, *Lupinus albus* had the highest root physiology scores, followed by *Cicer arietinum* > *Vicia faba* = *Glycine max* > *Zea mays* > *Triticum aestivum* = *Brassica napus* (**Figure 9**).

## Discussion

### Plant Growth, Biomass Distribution and Root Morphological Responses to Variable P Supply

This study revealed that the plant growth responses to P supply under variable soil conditions (AS vs. CS) varied among plant species with contrasting root properties (**Figure 1**). The fibrous-root species *Zea mays*, *Triticum aestivum* and *Brassica napus* had greater shoot growth responses to P supply than the tap-rooted legumes *Lupinus albus, Glycine max, Vicia faba*, and *Cicer arietinum* in both soil types (**Figure 2**). The results suggested that the fibrous-root species had strong dependence on external P application, but low dependence on inherent soil residual P with relatively low availability; in contrast, the tap-rooted legumes could acquire substantial amounts of residual P from soil, relying relatively little on fertilizer P application. A similar pattern was also found for shoot P content in the present study: the fibrous-root species *Zea mays*, *Triticum aestivum*, and *Brassica napus* had greater responses to P supply than the tap-rooted legumes *Lupinus albus*, *Glycine max*, *Vicia faba*, and *Cicer arietinum* in both soil types. Clearly, the responses of shoot dry weight, root/shoot ratio (except *Zea mays* in AS, **Figure 2**), and shoot P content (**Figure 6**) to P supply showed consistent results, compared with plant growth performance (**Figure 1**, **Supplementary Table S1**). Hence, plant growth responses to P supply under given soil conditions vary among plant species with contrasting root traits, indicating a potential strategy for modification of specific root traits to improve access to different P resources and thus enhance P acquisition by a given species (Shen et al., 2011, 2013).

The general response of shoot dry weight to P application was greater in CS than AS (*P* < 0.01), despite a variation among different plant species (Table S1). Phosphorus supply increased shoot biomass of all plant species except *Lupinus albus* in CS because this species is sensitive to CS as shown by leaf chlorosis (**Figure 1**, see also Kerley, 2000; Kerley and Huyghe, 2001). All other species (especially *Zea mays*, *Triticum aestivum*, *Brassica napus*, and *Cicer arietinum*) accumulated more shoot biomass in calcareous than AS (**Figures 2A,B**; see also Nuruzzaman et al., 2005; Pearse et al., 2007; Li et al., 2010; Rose et al., 2010) because plants could take up P from Ca complexes easier than from Fe and Al oxide complexes (Hinsinger, 2001; Veneklaas et al., 2003; Pearse et al., 2007). These effects could partially account for the increased biomass of plants (*Zea mays*, *Triticum aestivum*, *Brassica napus*, and *Cicer arietinum*) in CS compared with AS.

Many species distributed a greater proportion of total dry matter to root growth under P deficiency (Hill et al., 2006; Richardson et al., 2009). In the present study, cereals (*Zea mays* and *Triticum aestivum*) had higher root/shoot ratio than the legume species (*Lupinus albus*, *Glycine max*, *Vicia faba*, and *Cicer arietinum*), suggesting the former species allocated proportionally more biomass to roots in P-deficient soil. Also, the species with fibrous roots (particularly *Triticum aestivum* and *Brassica napus*) had relatively higher SRL compared with legume species (**Figures 3C,D**), indicating a smaller root diameter for fibrous root species. This means the fibrous-root species (*Zea mays*, *Triticum aestivum*, and *Brassica napus*, root diameter = 0.12 ± 0.04 mm, *n* = 48) have significantly thinner roots compared with the legume species (especially *Lupinus albus*, *Vicia faba*, and *Cicer arietinum*, root diameter = 0.24 ± 0.01 mm, *n* = 48) (data not shown).

Phosphorus deficiency significantly increased the SRL of *Zea mays* in CS and *Brassica napus* (but not of legume species) in AS (**Figure 3**). Even though an increase in SRL is not always a universal response to low P supply (Schroeder and Janos, 2005; Pang et al., 2010), increased production of relatively fine roots to create a large surface area of contact between roots and soil would be expected to enhance acquisition of P (see also Barber et al., 1963; Nuruzzaman et al., 2005; Pearse et al., 2006a, 2007; Calderón-Vázquez et al., 2009; Shen et al., 2011; Liu et al., 2016). The roots with small diameter were considered an efficient and economical means of increasing P acquisition (Eissenstat, 1992; Lynch, 2011, 2013). The reason for the increased SRL of *Zea mays* in AS and of *Brassica napus* in CS under P addition could be mainly due to low available P in soil, which appeared to have been quite severe in P-deficiency stress with low shoot P concentrations in this study (**Figure 6**). Hence, adding P alleviated the P stress, allowing plants to respond by increasing total root length and SRL.

Root/shoot ratio of legume species did not show a significant variation in response to P application, especially in AS, and quite a similar pattern was found for total root length and SRL. In particular, *Lupinus albus* had the smallest root/shoot ratio and total root length among all the species (**Figures 2** and **3**). These relatively small morphological responses of *Lupinus albus* to P application suggested that effective mobilization of rhizosphere P (physiological response) is more resource-efficient than extending roots (morphological response) to get P from far away (Shen et al., 2011). Indeed, cluster roots (**Supplementary Figure S1**) enhanced root exudation, thus enhancing the root physiological responses (Shen et al., 2005; Tang et al., 2013b).

In conclusion, the fibrous-root species (*Zea mays*, *Triticum aestivum*, and *Brassica napus*) showed larger root morphological plasticity as a way of increasing P acquisition to cope with variable P supply than the tap-rooted legumes (*Lupinus albus, Glycine max, Vicia faba*, and *Cicer arietinum*).

### Root Physiological Responses

The physiological and biochemical responses involved decreased shoot P concentration and content (**Figure 6**), enhanced internal P utilization and increased root exudation into the rhizosphere. Root exudation of acid phosphatase (APase) and carboxylates are considered important for P mobilization and acquisition (Raghothama, 1999; Vance et al., 2003; Lambers et al., 2006; Richardson et al., 2009). In the present study, APase activity and carboxylate content in the rhizosphere of *Lupinus albus* were significantly higher than in the other plant species, but *Lupinus albus* showed no significant difference between the P treatments in either soil (**Figure 4**). The reason could be related to cluster root formation in both P treatments, and there was no difference in the proportion of cluster root dry weight with respect to the whole root system (data not shown) in either P treatment, possibly because of a relatively small soil volume restricting the total amount of P present.

In the present study, the *Lupinus albus* shoot P concentration was <2.5 mg g^-1^ in AS and <1.5 mg g^-1^ in CS regardless of P treatments, which is lower than the critical level at or below which cluster root formation and citrate exudation would be significantly up-regulated according to our previous study (Li et al., 2008). This could partly account for the strong cluster root formation, citrate exudation and APase activity in the rhizosphere, which are regulated by low shoot P status as an internal systemic signal (Shen et al., 2005; Li et al., 2008; Shen et al., 2013; Tang et al., 2013a). Indeed, in the present study *Lupinus albus* efficiently acquired soil P through high exudation of carboxylates and acid phosphatases that modified rhizosphere chemistry, but this species exhibited a relatively low response to the applied fertilizer P; in contrast, the fibrous-root species with low capacity for root exudation of carboxylates and acid phosphatases showed a strong response to the applied P through altering the root morphological traits.

Other legume species (*Glycine max*, *Vicia faba*, and *Cicer arietinum*) showed higher or slightly higher activity of APase in the rhizosphere soil compared with the cereal species in the present study (**Figures 4A,B**) as well as in other studies (Tadano et al., 1993; Duff et al., 1994; Li et al., 2004; Nuruzzaman et al., 2006; Wang et al., 2008). Similarly, exudation of carboxylates was greater in the legume species, especially *Lupinus albus* and *Cicer arietinum*, compared with the fibrous-root species (**Figures 4C,D**; see also Ryan et al., 2001; Veneklaas et al., 2003; Li et al., 2007, 2010; Rose et al., 2010).

Phosphorus-utilization efficiency of different plant species was directly related to P concentration in plant tissues (Barker and Pilbeam, 2007); P starvation increased P-utilization efficiency as reported before (Rose et al., 2011). Therefore, the fibrous-root species (*Zea mays*, *Triticum aestivum*) coped with variable P supply through expanding the root absorption surface area to enhance spatial availability of P, but were less dependent on root exudation, compared with the legume species with intensive root exudation (Eissenstat, 1992; Ryan et al., 2001; Veneklaas et al., 2003; Pearse et al., 2006a, 2007; Rose et al., 2010). The results indicated a strategic variation in how different plant species enhance P acquisition by changing the coordination between root morphological and physiological traits.

### Root Morphological and Physiological Responses to Variable P Supply

Numerous studies compared the responses of plant species to various external P supply conditions. However, the conclusions of all these studies mainly focused on independent changes in either root morphological or physiological parameters, such as shoot biomass or carboxylate exudation (Hoffland et al., 1989a,b; Tadano et al., 1993; Neumann and Römheld, 1999; Nuruzzaman et al., 2005; Li et al., 2010; Rose et al., 2010; Vu et al., 2010; Maltais-Landry, 2015), or some correlation between these parameters (Shen et al., 2003; Watt and Evans, 2003; George et al., 2006; Pearse et al., 2007; Zhang et al., 2009). In this study, the PCA method was used as reported before (Tang et al., 2013b) to calculate the relative contribution of the root morphological or physiological response parameter scores to P acquisition, and then quantitatively evaluate the relationship between root morphological and physiological traits in response to P supply and soil types (**Figures 8** and **9**). *Zea mays* and *Triticum aestivum* had higher root morphology scores (strong response in root/shoot ratio, total root length and SRL; **Figures 2** and **3**) and low physiology scores (low exudation of APase and carboxylates and consequently low P-acquisition efficiency; **Figures 4** and **7**), exhibiting greater changes in the root morphology scores to P supply in the two soil types compared with the legume species.

*Brassica napus* had the lowest physiology scores, but exhibited large changes in the root morphology scores. Previous studies indicated that *Brassica napus* had a strong physiological response to acquiring P (Foehse and Jungk, 1983; Moorby et al., 1988; Hoffland et al., 1989a,b), but most of these studies were conducted at an early growth stage of *Brassica napus*. In the present study, plants were harvested at relatively mature stage, and the results showed *Brassica napus* mainly relied on the root morphological changes in response to variable P supply; indeed, at this growth stage some previous studies also reported low root exudation and high SRL in *Brassica napus* (Marschner et al., 2007; Pearse et al., 2007; Solaiman et al., 2007; Zhang et al., 2009).

*Lupinus albus* and *Cicer arietinum* showed the highest root physiology scores and the lowest root morphology scores among all plant species, probably related to strong root exudation and low root length despite formation of cluster roots in *Lupinus albus* (**Figures 3** and **4**). Furthermore, these two species also had strong variation in the root physiological scores to cope with P deficiency under different soil conditions. *Lupinus albus* had higher physiology scores in the –P compared with the +P treatment; *Cicer arietinum* had the opposite result. These patterns were related to higher exudation of carboxylates and acid phosphatase from cluster roots of *Lupinus albus* in the P-deficient treatment, and higher exudation of carboxylates by *Cicer arietinum* in the P-sufficient conditions (**Figure 4**, see also Tadano et al., 1993; Neumann et al., 1999; Yan et al., 2002; Shen et al., 2003; Vance et al., 2003; Veneklaas et al., 2003; Wouterlood et al., 2004; Lambers et al., 2006; Wang et al., 2007; Rose et al., 2010; Cheng et al., 2014). These results indicated that *Lupinus albus* and *Cicer arietinum* depended mainly on the physiological responses to variable P supply in AS and CS, emphasizing an exudation component of cluster roots. In contrast, *Glycine max* and *Vicia faba* roots showed changes in neither morphological nor physiological response to P supply. Taken together, the *Lupinus albus* and *Cicer arietinum* response to P deficiency mainly depended on the root physiological traits, whereas *Zea mays*, *Triticum aestivum*, *and Brassica napus* had the strong root morphological responses. *Glycine max* and *Vicia faba* showed a combination of root morphological and physiological responses. This knowledge is critical for manipulating root morphology and rhizosphere processes in a given species to enhance plant growth and P-acquisition efficiency.

## Conclusion

This study provided novel evidence of variable coordination and balance between root morphological and physiological responses to cope with P supply changes in different plant species, suggesting that the specific strategy could be developed to modify root morphological or physiological traits for a given plant species to increase P-acquisition and P-utilization efficiency. Further work on other morphological and physiological traits (e.g., root angles, root hairs, mycorrhizal colonization, P uptake) as well as on other plant species to underpin phylogenetic extrapolation is needed.

## Author Contributions

YL, HL, FZ, and JS designed the experiments; YL performed the experiments; YL, HT, and JS carried out data analysis and wrote the manuscript; ZR and WW contributed to the interpretation of data analyses and manuscript writing.

## Conflict of Interest Statement

The authors declare that the research was conducted in the absence of any commercial or financial relationships that could be construed as a potential conflict of interest.
