# Supplementary material for: Major Crop Species Show Differential Balance between Root Morphological and Physiological Responses to Variable Phosphorus Supply
Source: Front Plant Sci. 2016 Dec 21;7:1939. doi: 10.3389/fpls.2016.01939 (PMC5174099; doi:10.3389/fpls.2016.01939)
Supplement: Supplementary file 1 [file Table_1.DOC]

***Supplementary Material***

# **Major Crop Species Show Differential Balance between Root Morphological and Physiological Responses to Variable Phosphorus Supply**

**Yang Lyu, Hongliang Tang, Haigang Li, Fusuo Zhang, Zed Rengel, William R. Whalley, Jianbo Shen***

***Correspondence:** Prof. Jianbo Shen: jbshen@cau.edu.cn

**Table S1** Results of three-way ANOVA for shoot dry weight, root/shoot ratio, total root length, specific root length, acid phosphatase activity and carboxylates in the rhizosphere soil, shoot P concentration, shoot P content, P-acquisition efficiency and P-utilization efficiency of all species in two soil types with or without P addition. Significant P values are in bold.

| Parameters | Species | | Soil | | P | | Species × Soil | | Species × P | | Soil × P | | Species × Soil × P | |
| --- | --- | --- | --- | --- | --- | --- | --- | --- | --- | --- | --- | --- | --- | --- |
|  | *F*-value | *P*-value | *F*-value | *P*-value | *F*-value | *P*-value | *F*-value | *P*-value | *F*-value | *P*-value | *F*-value | *P*-value | *F*-value | *P*-value |
| Shoot dry weight | 338 | **< 0.001** | 400 | **< 0.001** | 1568 | **< 0.001** | 47 | **< 0.001** | 174 | **< 0.001** | 293 | **< 0.001** | 39 | **< 0.001** |
| Root/shoot ratio | 333 | **< 0.001** | 2.8 | 0.098 | 316 | **< 0.001** | 3.3 | **0.005** | 36 | **< 0.001** | 111 | **< 0.001** | 8.5 | **< 0.001** |
| Total root length | 169 | **< 0.001** | 33 | **< 0.001** | 539 | **< 0.001** | 64 | **< 0.001** | 96 | **< 0.001** | 0.03 | 0.873 | 46 | **< 0.001** |
| Specific root length | 312 | **< 0.001** | 2.7 | 0.102 | 1.6 | 0.216 | 13 | **< 0.001** | 1.7 | 0.142 | 19 | **< 0.001** | 30 | **< 0.001** |
| Acid phosphatase activity in the rhizosphere soil | 179 | **< 0.001** | 98 | **< 0.001** | 3.3 | 0.073 | 9.2 | **< 0.001** | 3.1 | **0.008** | 0.11 | 0.745 | 2.7 | **0.019** |
| Carboxylates in the rhizosphere soil | 76 | **< 0.001** | 91 | **< 0.001** | 6.8 | **0.011** | 25 | **< 0.001** | 4.2 | **< 0.001** | 3.9 | 0.052 | 0.71 | 0.641 |
| Shoot P concentration | 37 | **< 0.001** | 47 | **< 0.001** | 292 | **< 0.001** | 17 | **< 0.001** | 11 | **< 0.001** | 48 | **< 0.001** | 5.7 | **< 0.001** |
| Shoot P content | 173 | **< 0.001** | 735 | **< 0.001** | 2197 | **< 0.001** | 90 | **< 0.001** | 113 | **< 0.001** | 784 | **< 0.001** | 67 | **< 0.001** |
| P-acquisition efficiency | 42 | **< 0.001** | 2.7 | 0.102 | 60 | **< 0.001** | 12 | **< 0.001** | 0.65 | 0.691 | 24 | **< 0.001** | 3.2 | **0.008** |
| P-utilization efficiency | 134 | **< 0.001** | 87 | **< 0.001** | 605 | **< 0.001** | 22 | **< 0.001** | 20 | **< 0.001** | 90 | **< 0.001** | 26 | **< 0.001** |
