# Supplementary material for: Major Crop Species Show Differential Balance between Root Morphological and Physiological Responses to Variable Phosphorus Supply
Source: Front Plant Sci. 2016 Dec 21;7:1939. doi: 10.3389/fpls.2016.01939 (PMC5174099; doi:10.3389/fpls.2016.01939)
Supplement: Supplementary file 2 [file Table_2.DOC]

***Supplementary Material***

# **Major Crop Species Show Differential Balance between Root Morphological and Physiological Responses to Variable Phosphorus Supply**

**Yang Lyu, Hongliang Tang, Haigang Li, Fusuo Zhang, Zed Rengel, William R. Whalley, Jianbo Shen***

***Correspondence:** Prof. Jianbo Shen: jbshen@cau.edu.cn

**Table S2** Eigenvector scores of root morphological and physiological variables in the three main principal components (PC). Values are ranked based on the values of the first principal components. The two highest eigenvector scores for each PC are indicated in bold. Values in parentheses indicate a proportion of total variance accounted for by each PC.

| Morphological variables | | | |  | Physiological variables | | | |
| --- | --- | --- | --- | --- | --- | --- | --- | --- |
| Variables | PC 1  (45%) | PC 2  (33%) | PC 3  (15%) |  | Variables | PC 1  (35%) | PC 2  (28%) | PC 3  (22%) |
| Total root surface area | **0.355** | 0.09 | -0.17 |  | Malate concentration | **0.365** | 0.301 | 0.174 |
| Root biomass | **0.341** | -0.115 | -0.114 |  | Citrate concentration | **0.363** | 0.322 | 0.041 |
| Total root length | 0.316 | 0.212 | -0.147 |  | P-acquisition efficiency | 0.292 | **-0.423** | -0.073 |
| Root/shoot ratio | 0.128 | 0.12 | **1.011** |  | Acid phosphatase activity | 0.186 | -0.184 | **0.58** |
| Specific root length | -0.081 | **0.467** | -0.02 |  | Rhizosphere pH | -0.169 | -0.181 | **0.569** |
| Specific root surface area | -0.082 | **0.458** | **-0.171** |  | P-utilization efficiency | -0.245 | **0.407** | 0.278 |
