# Supplementary material for: Major Crop Species Show Differential Balance between Root Morphological and Physiological Responses to Variable Phosphorus Supply
Source: Front Plant Sci. 2016 Dec 21;7:1939. doi: 10.3389/fpls.2016.01939 (PMC5174099; doi:10.3389/fpls.2016.01939)

Supplementary Material

Major Crop Species Show Differential Balance between Root Morphological and Physiological Responses to Variable Phosphorus Supply

Yang Lyu, Hongliang Tang, Haigang Li, Fusuo Zhang, Zed Rengel, William R. Whalley, Jianbo Shen*

***Correspondence:** Prof. Jianbo Shen: jbshen@cau.edu.cn

Figure S1 Number (A) and fraction of root clusters (proportion of total root mass) (B) in *Lupinus albus* supplied with 0 (open bars) or 100 mg P kg^{-1} soil (closed bars) in acid or calcareous soil. Each value is the mean (+SE) of four replicates. Different letters denote significant differences between two soil types ($P \leq 0.05$). For each soil, asterisks indicate significant difference between the P treatments: * ($P \leq 0.05$).

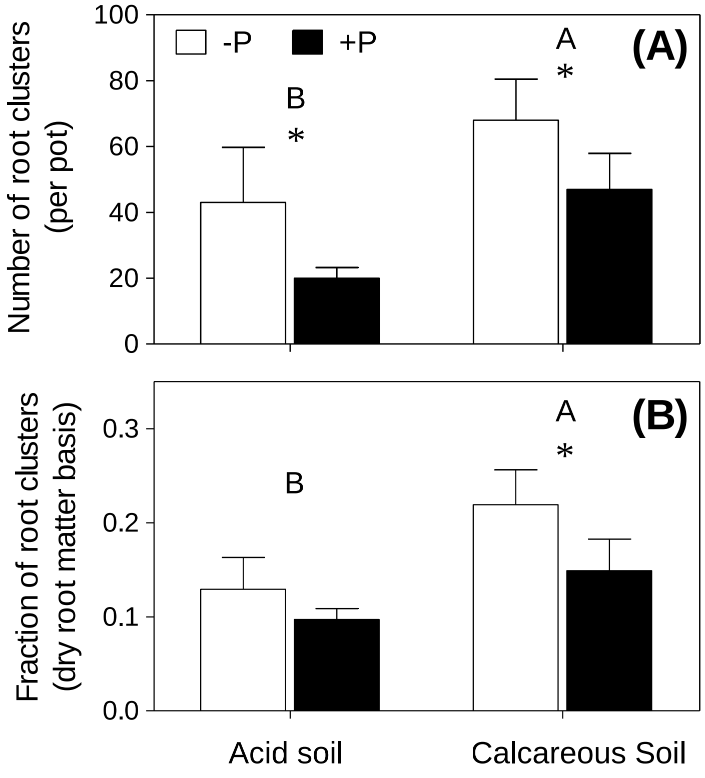

Supplement: Supplementary file 3 [file Image_1.PDF]
